# An Engineered Multimodular Enzybiotic against Methicillin-Resistant *Staphylococcus aureus*

**DOI:** 10.3390/life11121384

**Published:** 2021-12-10

**Authors:** Salim Manoharadas, Mohammad Altaf, Abdulwahed Fahad Alrefaei, Naushad Ahmad, Shaik Althaf Hussain, Basel F. Al-Rayes

**Affiliations:** 1 Department of Botany and Microbiology, College of Science, King Saud University, P.O. Box 2454, Riyadh 11451, Saudi Arabia; 2 Central Laboratory RM 63AA, College of Science, King Saud University, P.O. Box 2454, Riyadh 11451, Saudi Arabia; maltaf@ksu.edu.sa (M.A.); anaushad@ksu.edu.sa (N.A.); salthaf@ksu.edu.sa (S.A.H.); bfalrayes@ksu.edu.sa (B.F.A.-R.); 3 Department of Chemistry, College of Science, King Saud University, P.O. Box 2454, Riyadh 11451, Saudi Arabia; 4 Department of Zoology, College of Science, King Saud University, P.O. Box 2454, Riyadh 11451, Saudi Arabia; afrefaei@ksu.edu.sa

**Keywords:** enzybiotics, bacteriophage, MRSA, antibiotic-resistant bacteria, *Staphylococcus aureus*

## Abstract

Development of multidrug antibiotic resistance in bacteria is a predicament encountered worldwide. Researchers are in a constant hunt to develop effective antimicrobial agents to counter these dreadful pathogenic bacteria. Here we describe a chimerically engineered multimodular enzybiotic to treat a clinical isolate of methicillin-resistant *Staphylococcus aureus* (*S. aureus*). The cell wall binding domain of phage ϕ11 endolysin was replaced with a truncated and more potent cell wall binding domain from a completely unrelated protein from a different phage. The engineered enzybiotic showed strong activity against clinically relevant methicillin-resistant *Staphylococcus aureus*. In spite of a multimodular peptidoglycan cleaving catalytic domain, the engineered enzybiotic could not exhibit its activity against a veterinary isolate of *S. aureus*. Our studies point out that novel antimicrobial proteins can be genetically engineered. Moreover, the cell wall binding domain of the engineered protein is indispensable for a strong binding and stability of the proteins.

## 1. Introduction

Multidrug-resistant pathogens cause a serious threat to the health of both humans and animals. More than two million people are infected every year with conventional antibiotic-resistant bacteria in the United States alone [1]. Besides the fact of antibiotic resistance development, there is a problem of non-specific damage that the conventional antibiotics cause to commensal microbes, hence causing long-term harmful effects like secondary infections, obesity, asthma, and type I and II diabetes [2,3,4,5]. Therefore, it is an absolute necessity to develop specific therapeutics to target drug-resistant pathogens, without affecting the commensal microbiota. There is a new class of enzyme-based antibacterials, known as enzybiotics that could potentially replace antibiotics in the near future. Enzybiotics are generally safe, fast acting, highly specific and effective. In addition, there is a low probability of the development of bacterial resistance to enzybiotics. Furthermore, it has been shown that enzybiotics can be used alone or in combination with traditional antibiotics, generally reducing the effective concentration of the antibiotics [1,6]. Enzybiotics are generally derived from bacteriophage endolysins, which lyse the bacterial cells at the end of the lytic replicative cycle, facilitating the release of phage progenies. This feature of the endolysin has been exploited to kill Gram-positive pathogens, when added exogenously [7]. It has been shown that the exogenous addition of recombinant enzybiotics induces rapid osmotic lysis of Gram-positive bacteria, primarily through the degradation of accessible peptidoglycan. In vivo efficiency of various enzybiotics has been shown in animal models of human disease [7,8,9,10]. Recent studies have also pointed out that enzybiotics are effective against MRSA strains in wound infections [11]. In addition to wound infections, enzybiotics have also been shown to penetrate cells and eradicate intracellular antibiotic-resistant *S. aureus* pathogens [12].

Apart from the studies of enzybiotics on the treatment of pathogenic bacteria, enzybiotics have also been successfully used for food bio-preservation [13]. This is particularly important as multidrug-resistant bacteria can reach the food supply chain from farm to postharvest and processing such as fermentation, slaughtering, packaging and storage [14,15,16]. Another study where enzybiotics have been successfully used was in orthopedic device-related infections (ODRI) [17]. The most common bacteria associated with bone-related infections is the *Staphylococcus* species with an infection rate of ~66% [18,19]. The prevalence of MRSA often complicates the situation with biofilm formation on the surgical implants [20,21]. Two enzybiotics, M23 and GH15, were efficient in killing both planktonic and biofilm encased *S. aureus* strains associated with ODRI [17].

There are marked advantages of engineering enzybiotics against pathogenic bacteria. The engineering of enzybiotics, typically by replacing the cell wall binding domain making the lysin active against a larger number of bacterial strains. Apart from this, engineered enzybiotics also behave differently in comparison with their non-engineered counterparts. For instance, the natural endolysin (Pal) of the *pneumococcal* phage Dp-1 was show to be of intergeneric origin and displayed activity against Gram-positive bacteria [22]. However, part of the lysin was used for creating an engineered enzybiotic, known as artilysins. The engineered enzybiotic was able to lyze Gram-negative bacteria also in addition to Gram-positive bacteria [23,24]. The development of novel chimeric enzybiotics also provides help to determine the molecular evolution of genes. [25,26]. A detailed review was published in 2018 regarding the strategies employed and advantages of engineered phage-derived lytic enzymes in comparison to their parental counterparts [27].

In this study we investigated the antimicrobial potential of a chimeric enzybiotic against *S. aureus* including the clinical MRSA strain. Our idea was to compare the antimicrobial efficiency of two proteins, CA and CA100. Protein CA encompassed the CHAP and amidase residues from the *S. aureus* phage ϕ11 without the cell wall binding domain. CA100 on the other hand harbored the CHAP and amidase domain in the N-terminal and part of the cell wall binding domain from protein P17 from the *S. aureus* phage ϕ44AHJD. We tested the antimicrobial efficiency of both the proteins on three *S. aureus* strains: *S. aureus* 8325-4, clinical MRSA and *S. aureus* Rumba, a veterinary isolate. Our data suggest that newer, effective enzybiotics can be developed by replacing the native cell wall binding domain.

## 2. Materials and Methods

### 2.1. Bacterial Strains and Bacteriophages

The *S. aureus* strain Rumba, *S. aureus* 8325-4, clinical MRSA and *E. coli* strain KKH001 were used to study the effectiveness of the proteins. The *S. aureus* strain Rumba was a veterinary isolate from the udder of a cow named ‘Rumba’ with bovine mastitis. Clinical MRSA was obtained from King Khalid Hospital & Prince Sultan Center for Health Care, Al Kharj, Saudi Arabia. *S. aureus* 8325-4 is a routinely used lab strain. *E. coli* strain KKH001 was collected from Al Dawadmi General Hospital, Al Dawadmi, Saudi Arabia. *S. aureus* RN4220 was used for the propagation of phage ϕ11 and *S. aureus* B68 was used for the propagation of phage ϕ44AHJD. *S. aureus* B68, *S. aureus* Rumba and phage ϕ44AHJD was a gift from Prof. Dr. Udo Blaesi, MFPL, Vienna, Austria. *S. aureus* RN4220 and phage ϕ11 was a gift from Prof. Dr. Andreas Peschel, Universität Tübingen, Germany.

### 2.2. Growth of Bacteria and Bacteriophages

The bacteria were grown in LB media/agar (1% peptone, 1% NaCl, 0.5% yeast extract, Micromaster, Thane, Maharashtra, India) pH: 7.0. The bacteria were grown at 37 °C under shaking conditions (160 rpm) in an orbital shaking incubator until unless otherwise mentioned. For the propagation of bacteriophages, 50 µL of 1 × 10^7^ plaque-forming unit (PFU) of phage was added to 500 mL of corresponding host bacterial strain grown until an OD_600_ of 0.5. The phage-added bacterial culture was incubated for further 5 h at 37 °C, under shaking conditions. The culture was centrifuged at 12,000 rpm for 30 m at 4 °C to remove unaffected bacteria and debris. The enumeration of bacteriophage by plaque-forming unit assay was performed according to the standard protocol [28]. The supernatant was stored at 4 °C until further use.

### 2.3. Extraction of Phage DNA and PCR Amplification of Genes

The phage DNA was extracted according to the protocol described by [29] with modifications. Briefly, the phage was mixed with host bacteria and poured on an LB plate with top agar (0.75% agar), and the plates were incubated at 37 °C, overnight. The concentration of phage used was fixed as to produce a confluent lysis of the host bacterium. Five mL of SM buffer (0.58% NaCl, 0.2% MgSO_4_.6H_2_O, 5 mL of 1 M Tris-Cl pH 7.5) was added onto the plate and the plate was incubated at 4 °C, overnight to elute phages from the top layer. After recovery, the mixture was centrifuged at 5000 rpm for 5 m and the supernatant was filtered over 0.22 µM filter. Five hundred µL of the lysate was treated with 1 µL of DNase I (1 U/µL, Fermentas, Waltham, MA, USA) and 1 µL RNase A (10 mg/mL, Fermentas, Waltham, MA, USA) for 1.5 h at 37 °C. The reaction was stopped by adding 20 µL of 0.5M EDTA (Fluka, Muskegon, MI, USA). The phage protein capsid was digested with, 1.25 µL of Proteinase K (20 mg/mL Fermentas, Waltham, MA, USA) followed by incubation at 56 °C for 1.5 h. The phage DNA was precipitated with 99% ethanol at −70 °C, overnight, followed by wash with 70% ethanol. The pellet was air dried and resuspended in autoclaved distilled water. 

The primers used for the amplification of *CA* and *CA100* are listed in Table 1. A 2× Pfu mastermix (G-Biosciences, St. Louis, MO, USA) was used for the amplification of the genes. The genes were amplified using the conditions as shown (94 °C for 7 m, 94 °C for 1 m, 62 °C for 30 s, 72 °C for 1.5 m, 72 °C for 7 m). The PCR was allowed to run for 35 cycles. The PCR amplified product was checked on a 1% agarose gel and the size of the products were determined.

### 2.4. Cloning and Expression of Genes

The PCR amplification of gene CHAP-amidase was performed using primers 1 and 2 (Table 1). The PCR fragment was restricted with BamHI/KpnI and was cloned into the expression vector pQE30, restricted with BamHI/KpnI hence creating the plasmid pQE-CA. The 300 bp cell wall binding gene fragment from gene 17 of phage ϕ44AHJD was amplified with primers 1 and 3 (Table 1) and was restricted with KpnI/HindIII. The restricted fragment was cloned downstream of gene CHAP-amidase in the vector pQE-CA, creating the plasmid pQE30-CA100. The cloned genes were confirmed by sequencing to rule out any possibility of mutations. The cloned genes in pQE-CA and pQE-CA100 were induced by 0.5 mM IPTG after growing the bacterial strain BL21 DE3 to an OD_600_ of 0.5 at 22 °C, under shaking conditions. The induced cultures were further incubated at 22 °C, 160 rpm for 16 h. A small aliquot of the cells (100 µL) was checked for protein synthesis by resolving on a 12% SDS-PAGE gel. The cultures were centrifuged at 5000 rpm for 30 m and the pellet was stored at −70 °C until further use.

### 2.5. Purification and Refolding of the Synthesized Proteins

Denaturation purification of the synthesized proteins was performed using standard protocol (Qiagen, St. Louis, MO, USA) by Ni-NTA agarose column (G-Biosciences, St. Louis, MO, USA), with modifications. Briefly, the cell pellet was resuspended in 10 mL of native lysis buffer (0.69% NaH_2_PO_4_, 1.75% NaCl, 0.068% imidazole, pH 8.0), along with a pinch of chicken egg white lysozyme (Research Lab, Mumbai, Maharashtra, India). The resuspended cell pellet was incubated on ice for 30 m. Pulse sonication cycles (60% power, 5 s pulse, 5 s off; Biosafer, Zhichunli, Beijing, China) were performed for 5 m to completely destroy the cell pellet followed by 4 °C refrigerated centrifugation at 5000 rpm for 40 m. The pellet was washed three times in 10 mL of washing buffer (6% urea, 1% Triton X100, 1.3% NaH_2_PO_4_, 0.1% tris, 1.1%NaCl pH 8.0). The solubilization of the proteins was performed by resuspending the pellet in solubilization buffer (48% urea, 1.3% NaH_2_PO_4_, 0.1% tris, 1.1% NaCl, 0.65% imidazole pH 8.0) for 16 h at 20 °C in an orbital shaking incubator at 50 rpm. The solubilized proteins were centrifuged at 5000 rpm for 40 m and the supernatant was collected. Ni-NTA agarose beads (1.0 mL) were added to the supernatant and incubated for 3 h at room temperature. This was followed by the washing of the Ni-NTA agarose, and Ni-NTA bound proteins were eluted in elution buffer (48% urea, 1.3% NaH_2_PO_4_, 0.1% tris, 1.1% NaCl, 0.7% imidazole pH 8.0). The eluted proteins were checked for purity by resolving on a 12% agarose gel. The concentration of the proteins was estimated by nanodrop (Bio-Rad, Des Plaines, IL, USA). The denatured proteins were refolded back to their native confirmation by 1-fold rapid dilution in buffer A (50 mM tris-Cl, 9.6 mM NaCl, 0.4 mM KCl, 1 mM EDTA pH: 8.5). Rapid dilution of denaturated proteins has been shown to be effective for refolding of proteins, particularly membrane-associated receptors and ligands [30]. Briefly, 100 µL of the eluted protein was dropped into 900 µL of buffer A with constant stirring. The mixture was incubated at 4 °C for 2 h. Activity of the protein was assessed by live–dead staining of bacteria followed by treatment with proteins, with buffer A maintained as a negative control. 

### 2.6. Live–Dead Staining of Bacteria and Confocal Microscopy

The live–dead staining of bacterial cells was performed according to the protocol described by Manoharadas et al., 2021 [31]. Briefly, the staining for viable cells was performed by the addition of 5 μM of SYTO9 (ThermoFischer Scientific, Bedford, MA, USA), diluted in DMSO. The staining was performed for 10 m in dark conditions. After incubation, the cover slips were further washed extensively with 1× PBS and final rinsing in double distilled water. In order to stain for nonviable cells, propidium iodide (ThermoFischer Scientific, Bedford, MA, USA) was diluted in 2× SSC buffer (0.3 M NaCl, 0.03 M sodium citrate, pH 7.0) to a final concentration of 500 nm. The diluted propidium iodide was added to the cells. The staining was performed for 10 m, followed by washing with 1× PBS and final rinsing with double distilled water. The image was captured by confocal microscope (Zeiss, Jena, Germany) at an excitation/emission of 483/503 nm for SYTO9 and 535/617 nm for propidium iodide. The images were acquired on a Rolera Em-C2 camera with a 63× oil immersion objective (Zeiss, Jena, Germany). The acquired image was processed by the Zen lite software (Zeiss, Jena, Germany).

### 2.7. Cell Binding Activity of Proteins and Western Blotting

To check the binding activity of the proteins to the bacterial cells, 50 ng of either protein CA or CA100 was added to 1 × 10^7^ cells and the mixture was incubated for 10 m at 37 °C. The cells were pelleted by centrifugation and the cells were washed three times with 1× PBS. The cells were boiled and were resolved on an SDS-PAGE gel followed by Western blotting. The supernatant was precipitated by TCA and was also resolved on SDS-PAGE gel and subjected to Western blot analysis.

The Western transfer of the proteins from SDS gel onto the nitrocellulose membrane (Thermoscientific, Bedford, MA, USA) was performed by semi-dry Western blot apparatus (Bio-rad, Des Plaines, IL, USA), according to the standard protocol. The protein transferred nitrocellulose membrane was blocked in 5% milk in 1× TBST buffer for 1 h at room temperature. After 1 h, the blot was washed three times with 1× TBST buffer followed by the addition of primary antibody (mouse anti-his antibody, Abclonal, Woburn, MA, USA) diluted to a concentration of 1:2000 and incubated for 16 h at 16 °C. The blot was further washed three times with 1× TBST and secondary antibody (goat anti-mouse IgG linked to alkaline phosphatase, Elabscience, Houston, TX, USA) was added diluted to a concentration of 1:10,000). The blot was developed, after further washing steps in 1× TBST, by the addition of BCIP/NBT (G-Biosciences, St. Louis, MO, USA).

### 2.8. Software Used for Statistical Analysis of the Data

The data obtained were analyzed for graph preparation using Microsoft Excel (2010 version). Mean values and standard deviation were calculated for each set of values using Microsoft Excel (2010 version). Error bars in the graph represent standard deviation. The statistical significance (*p*-values) of the data values was assessed using Welch and Brown–Forsythe one-way Anova. The *p*-values ≥ 0.05 were not significant (represented on the graph as *) and the *p*-values ≤ 0.05 were determined as significant (represented on the graph as **). The structure of both the proteins was predicted using the Phyre program [32].

## 3. Results

### 3.1. Construction of CA and CA100

**Figure 1:**
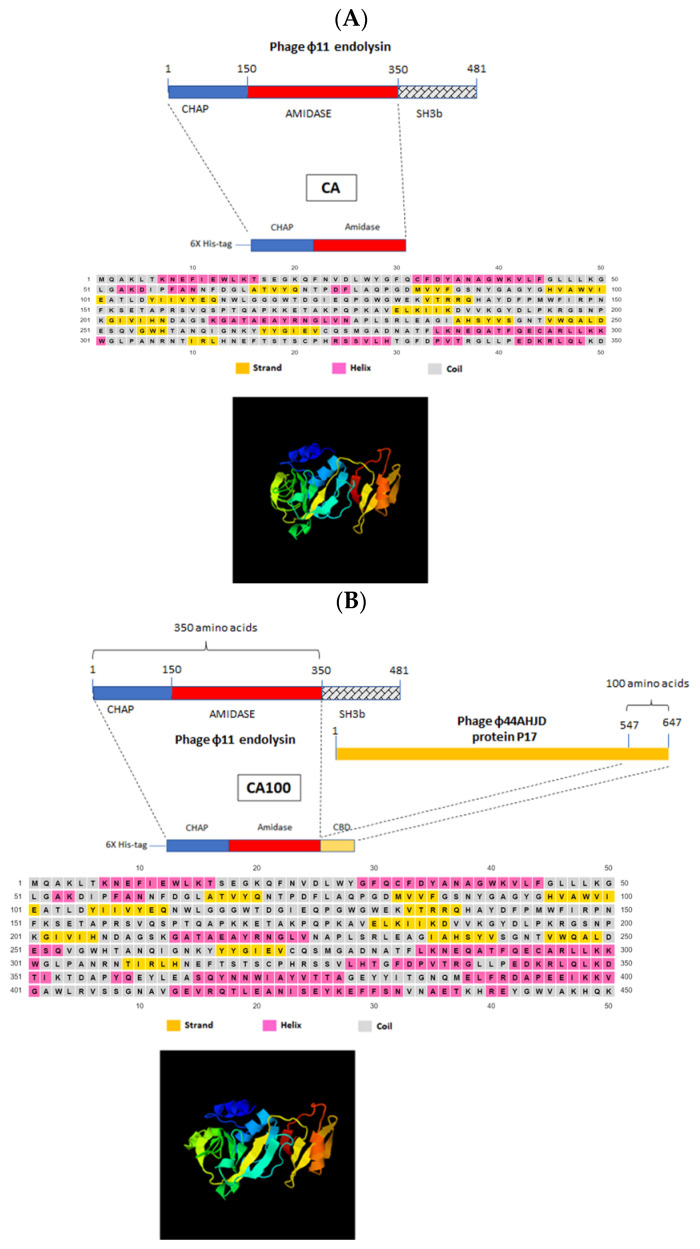
Modular design and structure of CA and CA100. (**A**) The parent protein (ϕ11 endolysin) from which the domains are used for the construction of protein CA is shown. The construction of protein CA, encompassing the CHAP and amidase domain with purification to be enabled by N-terminal 6x-histidine tag residues. The catalytic motifs CHAP and amidase are primarily composed of a coil structure (grey) with a few stretches of helix structure (pink). The C-terminal portion of protein CA also had a few helical stretches. The residues in yellow represent stranded structure. The predicted tertiary structure is also shown. (**B**) Protein CA100 had a 100 amino acid truncated cell wall binding domain (CBD) domain from protein 17 of phage ϕ44AHJD. The CBD predominantly comprised of a helix structure with a few stretches of coil structure. Stranded structure (yellow) was completely absent in the CBD. The predicted tertiary structure of CA100 is shown at the bottom.

The prediction of the structure of the catalytic domain (CHAP and amidase domain) of the endolysin from phage ϕ11 is shown in Figure 1A. The chimeric protein (CA100) was constructed by linking the CHAP-amidase domain of the endolysin of phage ϕ11 with 100 amino acid cell wall binding domain from protein P17 of the *S. aureus* phage ϕ44AHJD (Figure 1B). As seen in Figure 1, the C-terminal cell wall binding domain (CBD) linked to the CHAP-amidase domain consists primarily of helix structure. Interestingly, the CHAP-amidase protein without the CBD also had a long stretch of helix structure similar to the CBD from protein P17, invariably suggesting the capability of the CHAP-amidase domain in binding to the bacterial cells, albeit at a lower efficiency (Figure 1A). The CHAP domain is also primarily composed of a helix structure. However, the amidase domain is composed of a stranded structure. In our initial analysis, the CHAP domain alone did not show any activity. The amidase domain displayed activity against the *S. aureus* peptidoglycan. However, both the CHAP-amidase together, as found in the native endolysin, displayed a significantly higher peptidoglycan cleaving activity. Based on this initial analysis, we decided to work with the CHAP-amidase domain. The native C-terminal cell wall binding domain of the endolysin was replaced with the cell wall binding domain from protein 17 from phage ϕ44AHJD. It was earlier shown that the protein P17 was able to bind to a large number of *S. aureus* cells, suggesting a broader host range [33]. Hence, the linking of this cell wall binding domain to the catalytic domain presumably modifies the chimeric protein from the native phage ϕ11 endolysin. So, we decided to link the terminal 100 amino acid region to the CHAP-amidase domain from the endolysin of phage ϕ11.

### 3.2. Purification and Activity Checking of CA and CA100

**Figure 2:**
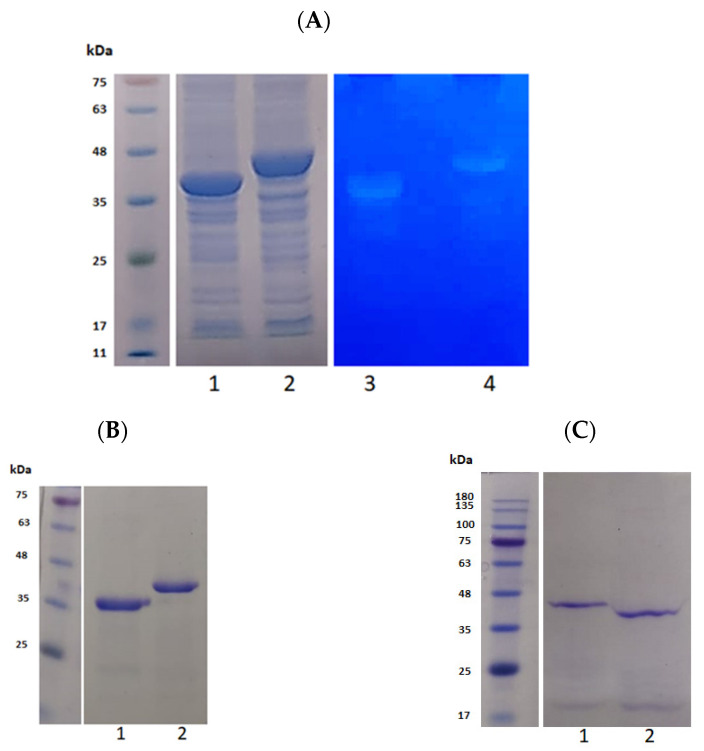
Expression and purification of CA and CA100. (**A**) The synthesized proteins CA and CA100 after IPTG induction of the genes are shown in lanes 1 and 2. The peptidoglycan cleaving activity of the proteins was checked on a zymogram with gel-embedded MRSA peptidoglycan shown on lanes 3 and 4. (**B**) The synthesized proteins were purified by Ni-NTA agarose. The proteins were ≈95% pure as judged on a SDS-PAGE gel. The purified proteins CA and CA100 are shown in lanes 1 and 2, respectively. (**C**) The denaturated proteins CA and CA100 were refolded back by rapid dilution in buffer A. The refolded proteins CA100 and CA used for further studies are shown in lanes 1 and 2, respectively.

Both gene CA and CA100 were cloned into pQE30 expression vector as BamHI/HindIII. The expression was performed by induction with 0.5 mM IPTG at an OD_600_ of 0.5. The expressed protein had N-terminal 6x histidine tag for purification with Ni-NTA column. The peptidoglycan cleaving activity of the expressed proteins were assessed on a zymogram with embedded clinical MRSA peptidoglycan. As seen in Figure 2A, the peptidoglycan cleaving activity of crude proteins CA and CA100 was detected on zymogram (lane 3 and 4), respectively. The expressed proteins were purified over the Ni-NTA column; the purity of the proteins was tested by loading onto SDS-acrylamide gel. As seen in Figure 2B, the proteins were ≈95% pure as visually analyzed from the gel. The proteins were purified after denaturation in 8% urea. Refolding of the proteins was performed by rapid dilution (1-fold) in buffer A. The refolded proteins were checked for stability in SDS- acrylamide gel. The refolded proteins were stable and no major degradation products were observed (Figure 2C). The concentration of the refolded proteins was estimated to be 0.4 mg/mL by nanodrop (Thermoscientific, Bedford, MA, USA).

### 3.3. CA100 Displays Activity against MRSA and S. aureus 8325-4

**Figure 3:**
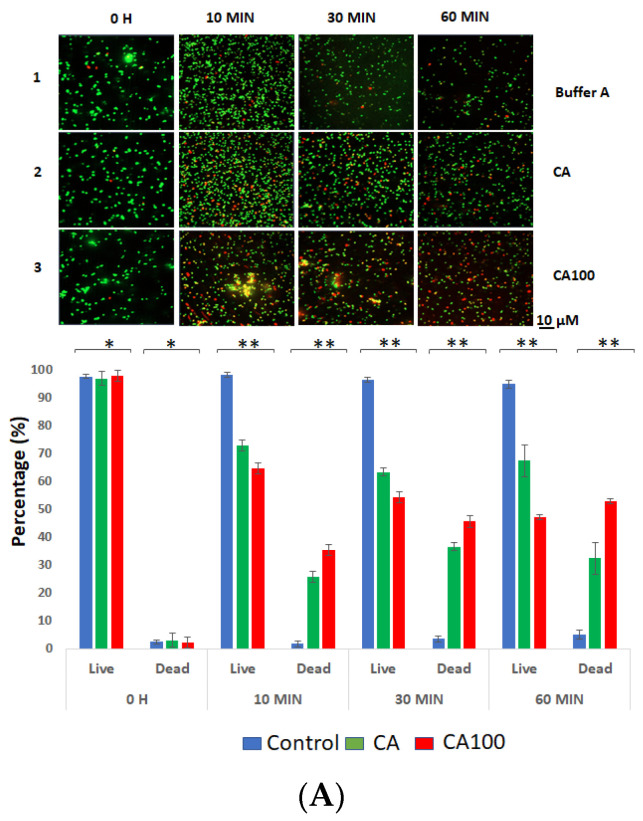

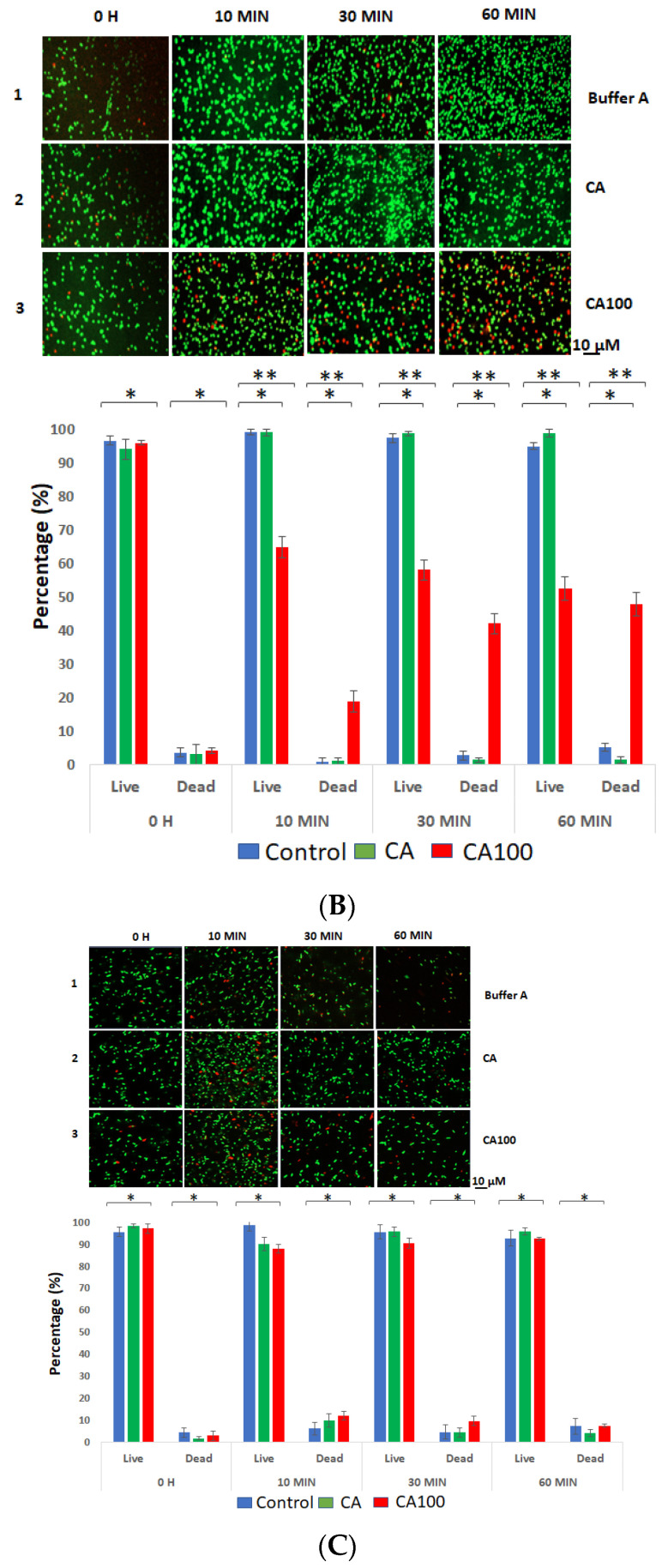

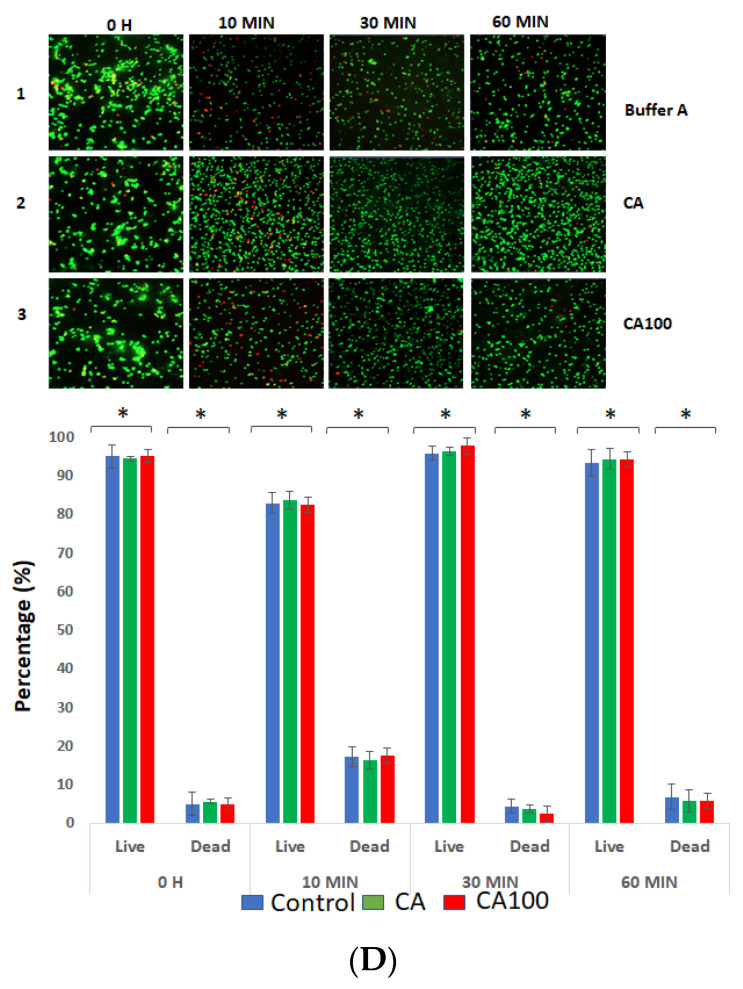
Live–dead staining of bacterial cells treated with proteins CA and CA100 (**A**) The treatment of *S. aureus* 8325-4 cells with protein CA caused a reduction of live cells by 23%, 63% and 67% within 10 m, 30 m and 60 m (lane 2). The reduction of live cells with CA100 amounted to 35%, 45% and 52% within 10 m, 30 m and 60 m (lane 3). The live cell in the buffer control was fixed to 100% at 0 h (lane 1). (**B**) The treatment of clinical MRSA cells with protein CA did not cause any reduction of live cells until 60 m in comparison with the buffer control (lane 2 vs. lane 1). The treatment of MRSA cells with CA100 caused a reduction in live cells amounting to 18%, 42% and 47% within 10 m, 30 m and 60 m (lane 3). (**C**) No reduction in live cells of clinical *E. coli* KKH001, in comparison to the control was seen within the tested time frame (lane 1 vs lane 2 vs. lane 3). (**D**) Treatment of *S. aureus* Rumba with CA and CA100 did not cause any visible reduction of live cells in comparison with the buffer control within the treated time (lane 1 vs. lane 2 vs. lane 3). The experiment was performed in triplicate and the error bars representing standard deviation are shown. Statistical significance level was calculated using one-way ANOVA (Welch and Brown–Forsythe) (* *p* ≥ 0.05; ** *p* ≤ 0.05).

To test the activity of the refolded proteins against *S. aureus* strains, 1 × 10^7^ cells were resuspended in buffer A (pH 8.5). Ten micrograms of the purified and refolded proteins, CA and CA100 were added to the resuspended cells. The mixture was incubated at 37 °C for 1 h. The live and dead cells following the treatment were assessed by confocal microscopy. The protein CA100 displayed activity against *S. aureus* 8325-4 (Figure 3A) and clinical MRSA cells (Figure 3B). In the case of *S. aureus* 8325-4, the number of CA-treated dead cells was 25% within 10 m. The number further dropped to 35% within 30 m of treatment and the number stayed almost the same until 60 m of treatment (Figure 3A). However, in the case of *S. aureus* 8325-4 cells treated with CA100, the number of dead cells was 35% within 10 m of treatment. The number of dead cells increased to 45% within 30 m and at the end of 60 m, 52% of *S. aureus* 8325-4 cells was observed to be dead (Figure 3A).

The treatment of the clinical MRSA strain with proteins CA and CA100 was interesting as protein CA did not show any activity against the MRSA strains until 60 m of treatment. However, the number of MRSA cells treated with CA100 displayed activity as 18% of dead cells were observed at 10 m of treatment. These values further reduced to 42% and 47% within 30 m and 60 m of treatment (Figure 3B). We further tested these proteins (CA and CA100) against a clinical *E. coli* isolate (KKH001) (Figure 3C) and veterinary isolate of *S. aureus* (Rumba) (Figure 3D). The *E. coli* KKH001 was maintained as a negative control as the outer LPS membrane was expected to prevent the access of the exolysins to the peptidoglycan. As seen in Figure 3C, neither of the two proteins was active against *E. coli* KKH001 until 60 m of treatment. Interestingly, the veterinary isolate, *S. aureus* Rumba was also not affected by both the proteins (Figure 3D), presumably suggesting that varied peptidoglycan chemistry exists in *S. aureus* Rumba.

### 3.4. Spectrophotometric Analysis of the Protein Treated Cells

**Figure 4:**
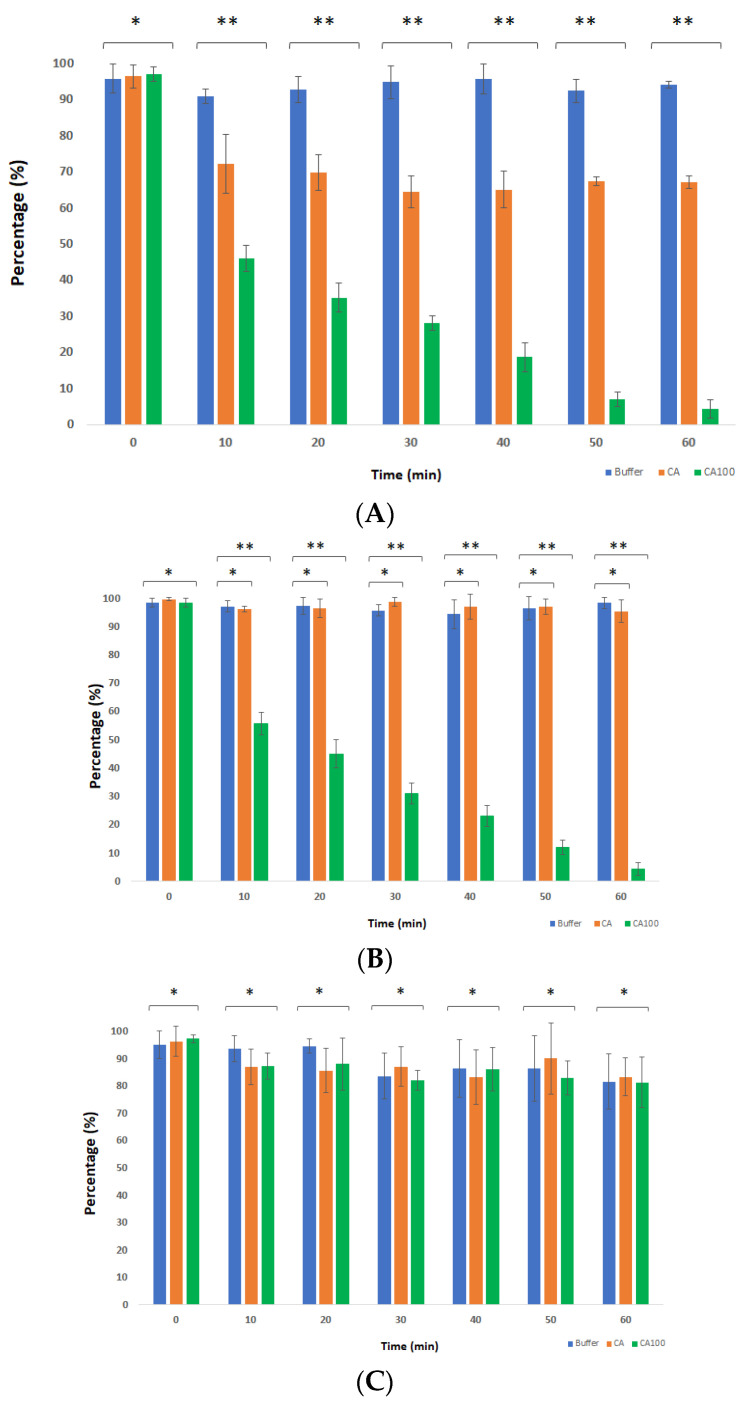

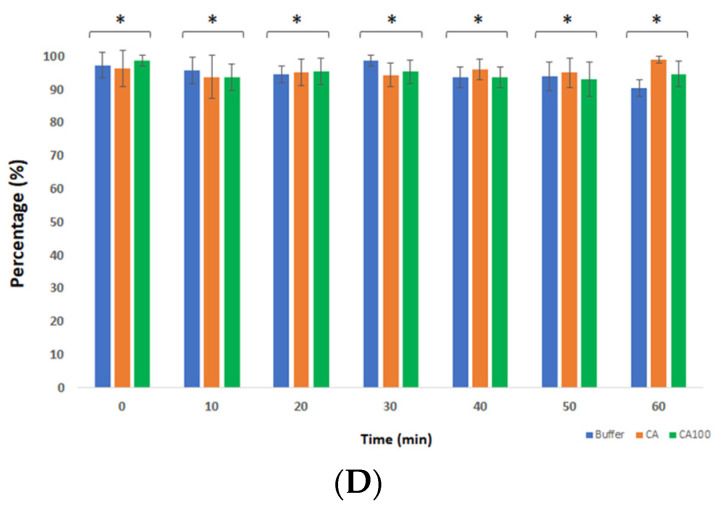
Spectrophotometric measurement analysis of the treated cells. (**A**) The reduction of cell density of *S. aureus* 8325-4 was measured spectrophotometrically at OD_600_. A decline in the cell density was seen in the CA treated sample amounting to 28% within 10 m and the percentage of reduction saturating at around 35% until 60 m of treatment. The reduction in live cell density was sharper in samples treated with CA100 with 54% reduction in 10 m (orange bar). The reduction was more prominent in the further tested time frame with the intensity dropping by more than 95% at the end of 60 m (green bar). Buffer control was set at 100% at 0 h for comparison (blue bar). (**B**) The treatment of MRSA strain with CA did not show any marked reduction in cell density in comparison with the control (orange bar vs. blue bar). The CA100 was effective in reducing the cell density with values dropping by 45% within 10 m of treatment. The cell density further reduced at the following time points with more than 95% reduction within 60 m of treatment (green bar). (**C**) No reduction in cell density was observed with either of the protein-treated *E. coli* KKH001 samples until 60 m (blue bar vs. orange bar vs. green bar). (**D**) Similar to *E. coli* KKH001, CA- and CA100-treated *S. aureus* Rumba cells did not show any reduction in cell density until 60 m following treatment (blue bar vs. orange bar vs. green bar). The experiment was performed in triplicate and the error bars represent standard deviation. The statistical significance level was calculated using one-way ANOVA (Brown–Forsythe and Welch) (* *p* ≥ 0.05; ** *p* ≤ 0.05).

In our previous experiment described in Section 3.3, we used the live–dead assay to determine the percentage of live and dead cells, which was observed by confocal microscopy. However, it was shown earlier that a localized rupture of the peptidoglycan leads to the loss of turgor pressure within the bacterial cell, leading to lysis and immediate death of the bacterium [5]. In the case of the lysis of bacterium, the genetic material would be leaked out, leaving only the debris. This would leave the propidium iodide unable to bind to the cellular DNA in live–dead staining. In order to have a direct inference about the activity of the proteins CA and CA100 on target bacteria, we measured the OD_600_ of the bacterial cells at different time points after treatment with the proteins. Similar to what was observed with the live–dead staining, the OD_600_ of *S. aureus* 8325-4 cells dropped by 28% within 10 m after treatment with protein CA. No major decrease in the OD_600_ was observed at further time points with the final value reaching 33% at the end of 60 m after treatment (Figure 4A; orange bars). In case of the treatment with CA100, the OD_600_ of *S. aureus* 8325-4 significantly reduced over time. A major drop of 54% was observed after 10 m following treatment of cells with the CA100 protein. The OD_600_ further reduced by 93% and 96% at the end of 50 m and 60 m, respectively (Figure 4A; green bars). This clearly states the antimicrobial activity of CA100 against *S. aureus* 8325-4.

The treatment of MRSA cells with proteins CA and CA100 drew a different picture in comparison with *S. aureus* 8325-4. No activity was seen with protein CA against MRSA (Figure 4B; orange bars). However, CA100 displayed activity against MRSA with the OD_600_ dropping by 55% within 10 m of treatment (Figure 4B; green bars). The drop in OD_600_ was consistent over time with only 4% of intact cells remaining after 60 m of treatment (Figure 4B; green bars).

In the live/dead experiment, no activity was seen for both the proteins CA and CA100 against *S. aureus* Rumba and *E. coli* KKH001. We wanted to test the effect that CA and CA100 had against both these strains spectrophotometrically. As seen in Figure 4C,D, no decrease in OD_600_ was noticed following treatment of *E. coli* KKH001 and *S. aureus* Rumba, respectively, with proteins CA and CA100.

### 3.5. Cell Wall Binding of the Proteins Determine Activity

**Figure 5:**
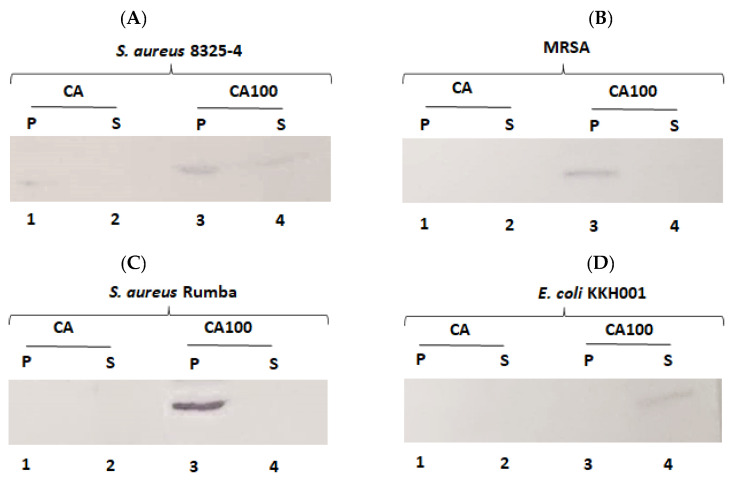
Assessment of cell wall binding ability of proteins CA and CA100. (**A**) The binding assay with *S. aureus* 8325-4 was performed with proteins CA and CA100. A weak binding of the protein CA was seen (lane 1). No protein was seen in the supernatant fraction (lane 2). A predominant amount of protein CA100 was bound to the cells (lane 3) and only a very small percentage was seen in the supernatant fraction (lane 4). (**B**) The protein CA100 was able to bind strongly to MRSA cells with almost complete protein CA100 bound to the cell pellet (lane 3). No protein was seen on the unbound supernatant fraction (lane 4). No cell wall binding was seen with protein CA (lane 2), neither was the protein found in the unbound supernatant fraction (lane 1). (**C**) A strong binding of CA100 was seen towards *S. aureus* Rumba with almost complete amount of the protein bound to the cell pellet (lane 3) and no protein was seen in the supernatant fraction (lane 4). Protein CA was neither found to be bound to the cell pellet (lane 1) nor found in the supernatant fraction (lane 2). (**D**) Neither protein, CA nor CA100, was able to bind to *E. coli* KKH001 (lane 1 and lane 3). The unbound CA100 protein was detected in the unbound supernatant fraction (lane 4). Protein CA was not detected in the supernatant fraction as well (lane 2).

We wanted to further test the cell wall binding of the proteins CA and CA100 to bacterial cells. We were particularly interested to see the binding of the proteins to *S. aureus* Rumba, as we did not observe any activity upon treatment with the proteins. Protein CA was found to be partially active against *S. aureus* 8325-4. We next tested the binding of the CA and CA100 proteins towards *S. aureus* 8325-4. A faint band of the cell pellet bound protein CA was seen after Western blotting. No protein was found in the supernatant fraction (Figure 5A; lane 1, 2). In contrast with CA, a strong pellet binding was observed with protein CA100. No complete (100%) binding was also observed with CA100 to the cell pellet, as a minor fraction of protein CA100 was also seen in the supernatant (Figure 5B; lane 3, 4). However, this pellet binding of CA100 was sufficient to exert a strong antibacterial activity.

The protein CA did not bind to the MRSA strain. No CA protein was found in either the pellet or supernatant fraction (Figure 5B; lane 1, 2). In contrast, CA100 protein bound strongly to the MRSA and the pellet bound protein was detected by Western blot (Figure 5B; lane 3, 4). This finding could answer why no activity was seen when CA protein was added to MRSA cells. The absence of CA protein in the supernatant also suggests that the protein may be degraded.

We did not observe any activity against *S. aureus* Rumba with either CA or CA100. We hypothesized that this could be because of the inability of the proteins to bind to the bacteria. To check this, CA or CA100 was tested for binding to *S. aureus* Rumba. Protein CA was found not to bind to the bacteria. No CA protein was found either in a pellet or supernatant fraction (Figure 5C; lane 1, 2). In contrast to our initial hypothesis, the CA100 protein was found to strongly bind to *S. aureus* Rumba (Figure 5C; lane 3). This result was interesting, as the protein CA100 could not exert antibacterial activity, in spite of its strong interaction with *S. aureus* Rumba. No CA100 protein was found in the unbound supernatant fraction (Figure 5C; lane 3), suggesting that most of the protein was bound to the cell pellet.

We also tested the binding of the proteins to the Gram-negative *E. coli* KKH001. As expected, neither CA nor CA100 was able to bind to the bacteria (Figure 5D; lane 1, 3). Protein CA was also not present in the unbound supernatant fraction (Figure 5D; lane 2). Protein CA100 was found in the supernatant fraction, confirming the inability of CA100 to bind to *E. coli* KKH001 (Figure 5D; lane 4).

### 3.6. Spot Analysis Shows the Activity of CA and CA100 

**Figure 6:**
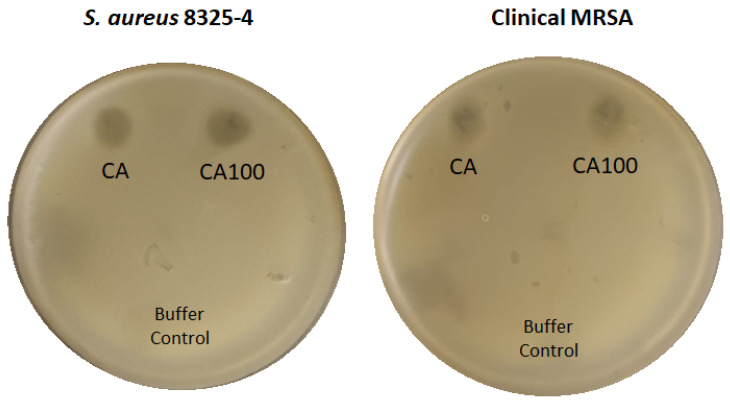
Spot analysis to determine activity of proteins against immovable cells. Proteins CA and CA100 (5 µL) were spotted on a lawn of *S. aureus* 8325-4 and MRSA. Buffer A was also spotted as a negative control. The plates were incubated at 37 °C for 16 h. Both CA and CA100 displayed antimicrobial activity against both the tested strains. The activity can be seen as a spot where no growth of bacteria was seen. No activity was seen in the region where buffer control was spotted.

Earlier experiments showed that CA100 was efficient against *S. aureus* 8325-4 and the clinical MRSA strain in liquid media. The CA protein however displayed nominal activity against *S. aureus* 8325-4 and showed no major activity against MRSA. In order to check the activity of protein CA and CA100 on solid media-grown bacteria, we did a spot analysis of both the proteins on *S. aureus* 8325-4 and MRSA. This work was performed particularly to assess the ability of the proteins in countering immovable bacteria especially in biofilm settings. Five µL of both the proteins (2 µg/µL) was spotted on a lawn of either *S. aureus* 8325-4 or MRSA. Both CA and CA100 were able to display activity against both the bacteria (Figure 6). It was interesting that CA was also active against MRSA in solid agar plates. The inability of CA to bind to MRSA was the factor that inhibited its activity in liquid suspension. As expected, CA100 shows activity against both the tested strains. Since no activity was observed with CA100 against *S. aureus* Rumba in spite of its strong binding, it was not tested on solid media. Gram-negative *E. coli* KKH001 have an outer LPS membrane that refrain the access of the exolysins to the peptidoglycan, hence was not tested on the solid media.

## 4. Discussion

There is an emergence of antibiotic-resistant bacteria with the list increasing rapidly over last few years and lesser new antibiotics are in the pipeline. This scenario has led to an intense need for novel types of antibiotics, to prevent public health crises [34]. Canonical antibiotics usually target critical bacterial processes like nucleic acid and protein synthesis, metabolic pathways or building of the outer cell envelope. Bacteria however overcome these challenges by either mutating the antibiotic target, inactivating the antibiotics or pumping them out using efflux pumps [35].

Bacteriophage-encoded lysins began to be used as a rapid and specific treatment to kill pathogenic bacteria 20 years ago [7]. The usage of purified phage lysins exogenously against pathogenic bacteria was called ‘enzybiotics’. One among the marked advantages of enzybiotics is that they target specific bonds in the bacterial peptidoglycan. Mutations in bacterial peptidoglycan often lead to compromising fitness in bacteria, hence, the emergence of bacterial resistance is highly unlikely [36,37]. Enzybiotics have been shown to be mostly active against Gram-positive bacteria and relatively unaffected Gram-negative bacteria, owing to the presence of an outer lipopolysaccharide membrane, which would act as a permeability barrier rendering inaccessibility to the lysin from reaching the bacterial peptidoglycan. In recent years, enzybiotics has been proposed as an important antimicrobial regime for containing multidrug-resistant Gram-positive bacteria [38,39,40,41]. *Staphylococcus aureus* has been a major nosocomial pathogen, associated with various infections like pneumonia, osteomyelitis and meningitis [42,43]. Furthermore, the development of antibiotic resistance by *S. aureus* has posed additional threats in hospitals [44]. 

We have devoted this study to the development of a chimeric enzybiotic against *S. aureus* strains. The strategy was to combine the catalytic domain of the endolysin from phage ϕ11 with the cell wall binding domain from protein 17 of phage ϕ44AHJD. The native catalytic domain comprises of the CHAP domain and the amidase domain. Both the domains were kept intact as removal of either of the domains compromised the activity of the protein. The native cell wall binding domain was replaced with the truncated cell wall binding domain from protein 17 of phage ϕ44AHJD, comprising of 100 amino acids. Earlier studies have pointed out that protein 17 from phage ϕ44AHJD was able to efficiently bind to a large number of *S. aureus* strains [33]. Protein 17 is a minor structural protein of phage ϕ44AHJD [45]. Moreover, the cell wall binding domain in protein 17 was proposed harbored within the C-terminal 25 amino acid region [6]. In our study, we basically compared the activity of two recombinantly synthesized proteins: CA and CA100. Protein CA comprised only of the catalytic domains, CHAP and amidase in tandem. CA100 however had an additional truncated cell wall binding domain downstream of the catalytic domains.

As is similar with the recombinant expression of other endolysins, proteins CA and CA100 were also expressed in inclusion bodies. Denaturation purification by solubilization of inclusion bodies by urea, followed by refolding of the proteins by rapid dilution to native confirmation was employed to purify the proteins. However, one disadvantage associated with rapid dilution was that the final concentration of proteins was reduced by 10 times. To counter this, we concentrated the Ni-NTA eluted proteins by Spin X concentrating columns (Corning, Corning, NY, USA), before rapid dialysis. The diluted and refolded proteins were further concentrated by Spin X columns. The activity of the refolded proteins was assessed by live–dead staining of bacterial cells after treatment with proteins.

Both the proteins CA and CA100 were found to be active against *S. aureus* 8325-4, however at variance. Protein CA was only partially active against *S. aureus* 8325-4, with 32% reduction of live cells within 60 m of treatment. However, protein CA100 was significantly more active against *S. aureus* 8325-4, with 52% reduction in live cells within 60 m of treatment. In the case of the clinical MRSA strain, only CA100 was found to be active with 47% reduction in live cells. Both the proteins were not active against the veterinary isolate *S. aureus* Rumba and clinical *E. coli* strain KKH001. These data were based on the staining of cells with SYTO9 dye and propidium iodide, to differentiate live and dead cells. In the case of Gram-positive bacteria, the peptidoglycan functions as the major structural component of the cell, supporting an internal turgor pressure of 20–50 atmospheres [30,46,47,48]. Any breach or rupture of the peptidoglycan layer results in osmotic lysis and cell death of the bacterium, similar to the phage progeny release during the culmination of the lytic infective cycle [8]. This bacterial rupture possibly leaks out the nucleic acids, hence live–dead staining does not provide a direct inference of the dead cells after treatment with the proteins. In order to have a direct assessment of the activity of the proteins on bacterial cells, spectrophotometric reduction assay of the cells was performed after treatment. The CA100 protein caused a 96% reduction in clinical MRSA cells within 60 m of treatment. As observed earlier, the CA protein was found not to be active against MRSA. The treatment of *S. aureus* 8325 with the CA and CA100 proteins showed a decline of live cells by 33% and 96%, respectively, within 60 m of treatment. Similar to the observation with live–dead staining, none of the proteins were active against *S. aureus* Rumba and *E. coli* KKH001. We hypothesized that the inactivity of the proteins against *S. aureus* Rumba could be because of the inability of the proteins to bind to the bacteria. To confirm this, we did a binding assay of the proteins towards the bacterial strains. Interestingly, CA100 was able to bind strongly to *S. aureus* Rumba. In addition, CA100 was also able to bind to MRSA and *S. aureus* 8325-4. As expected, CA100 did not bind to *E. coli* KKH001. Another important observation was that the CA protein was only able to partially bind to *S. aureus* 8325-4 and not to any other tested strains. However, CA was also not detected in the supernatant fraction stating that the cell wall binding domain in CA100 also presumably stabilizes the protein in addition to its cell wall binding function. Since we observed a weak binding of the CA protein to *S. aureus* 8325-4, we hypothesized that CA could exert its activity to the full potential in immovable cells. Spotting assay of proteins CA and CA100 was performed on a lawn of *S. aureus* Rumba and *S. aureus* 8325-4. Spot analysis was particularly carried out to know if the CA protein could exert activity against immovable *S. aureus* 8325-4 and MRSA, as cell wall binding domain is mostly necessary to bind the protein tightly to the bacterial cell in a liquid or moving culture. As hypothesized, both the proteins showed antibacterial activity on a spot test, further stating that the cell wall binding domain is indispensable for potent activity and stability of the constructed proteins.

Domain replacement strategy has been a forerunner in the creation of chimeric and novel enzybiotics in the recent decade. Apart from domain replacement, truncation and other modifications have been successfully tested to create more potential proteins against Gram-positive pathogens. In a recent study, a recombinant version of lysostaphin, LYSSTAPH-S and LYSDERM-S was created by gene sequence optimization and truncation of the pre-domain, to create a more effective protein that could be expressed and purified from *E. coli* [11]. In an earlier study, lysibodies were created by fusing cell wall binding domains from phage endolysins to IgG Fc, thereby creating novel therapeutic antibodies. This study also depicts the importance and specificity of the cell wall binding domain of phage lysins [49]. Engineering of phage endolysins also shows several advantages in comparison with their parental counterparts. In the activity level, the engineered phage lysins display an enhanced killing of bacteria in various growth conditions, thereby increasing the natural bactericidal spectrum [27]. The engineered lysins may also display increased solubility and subsequent stability at the production level. The increase in the catalytic activity of a modified phage endolysin was shown in a study, where a domain swapping or combinational approach was performed between the catalytic and cell wall binding domain from two *Listeria monocytogenes* phage endolysins, Ply118 and PlyPSA. A threefold higher activity against *Listeria* serovars was displayed by one of the engineered endolysins, EAD118_III_CBDPSA, in comparison with the parental lysin, PlyPSA [50]. The solubility of the phage lysins was improved by engineering of the endolysins to include a more soluble cell wall binding domain instead of the native cell wall binding domain. For instance, the chimeric endolysin, ClyS was composed of the catalytic domain from the endolysin (PlyTW) of *S. aureus* phage Twort and the cell wall binding domain from the endolysin of *S. aureus* phage phiNM3. The engineered endolysin ClyS displayed high solubility in production analysis. In addition, ClyS displayed a broad spectrum of activity against several *Staphylococcus* species both in vitro and in vivo [51,52].

We too have aimed to create a chimeric endolysin (CA100) with enhanced specificity and solubility. In our initial studies, the native endolysin of phage ϕ11, encompassing the catalytic and cell wall binding domain could not be purified to homogeneity owing to solubility problem (data not shown). The engineered protein, CA100, however was better soluble and displayed activity against the clinical MRSA strain.

## 5. Conclusions

Enzybiotics is an ever-emerging field to counter multidrug-resistant bacterial pathogens. With a rapid emergence of antibiotic-resistant strains and lack of new antibiotics, enzybiotics could play a pivotal role in antimicrobial therapy in the near future. Herein, we replaced the cell wall binding domain of the endolysin from phage ϕ11 with a truncated cell wall binding domain from a different phage. The engineered protein was active against the clinical MRSA strain. Our results also underline the importance of cell wall binding domain not only in specific binding, but also in providing stability for the recombinant protein.

## Figures and Tables

**Table 1:** Primers and plasmids used in this study.

Gene Amplified	Primer Sequences	Size (bp)
*CHAP-amidase*	Forward primer: 5′CTCAAGGATCCATGCAAGCAAAATTAACT3′	1050
*CHAP-amidase*	Reverse primer: 5′CATAGGTACCGTAGTCTTTAAGTTGCAACC3′	1050
*CBD100*	Forward primer: 5′CATAGGTACCATCAAAACTGACGCACCATAT3′	300
*CBD100*	Reverse primer: 5′CAGGAAGCTTCTATTTTTGATGTTTTGCTACC3′	300
Plasmid name	Notes on the plasmid	Synthesized protein upon IPTG induction
pQE30	Expression vector. Lac promoter induced with IPTG. Synthesized proteins have a 6X His-tag for purification.	NA
pQE-CA	The *CHAP-amidase* gene from phage ϕ11 cloned as BamHI/KpnI into pQE30 vector.	CHAP-amidase 38 kDa
pQE-CA100	The 300 bp cell wall binding gene from protein 17 of phage ϕ44AHJD was cloned as KpnI/HindIII into pQE-CA.	CHAP-amidase + Cell wall binding domain. 41 kDa

## Data Availability

The data presented in this study are available on reasonable request from the corresponding author.
